# Ghrelin Stimulates Endothelial Cells Angiogenesis through Extracellular Regulated Protein Kinases (ERK) Signaling Pathway

**DOI:** 10.3390/ijms19092530

**Published:** 2018-08-26

**Authors:** Jun Wang, Lin He, Bahetiyaer Huwatibieke, Lingchao Liu, He Lan, Jing Zhao, Yin Li, Weizhen Zhang

**Affiliations:** 1 Department of Physiology and Pathophysiology, School of Basic Medical Sciences, Peking University Health Science Center, Key Laboratory of Molecular Cardiovascular Science, Ministry of Education, Beijing 100191, China; wangjun_901016@sina.com (J.W.); tafcalgen@163.com (B.H.); liulingchao@bjmu.edu.cn (L.L.); pujingle@163.com (J.Z.); weizhenzhang@bjmu.edu.cn (W.Z.); 2 Department of Biochemistry and Molecular Biology, School of Basic Medical Sciences, Peking University Health Science Center, Key Laboratory of Carcinogenesis and Translational Research (Ministry of Education), Beijing 100191, China; helin910329@126.com; 3 Department of Clinical Laboratory, Capital Medical University, Beijing 100053, China; bylanhe08@126.com

**Keywords:** ghrelin, endothelial cells, angiogenesis, migration, ERK

## Abstract

Adipose tissue is hyper-vascularized. Vessels in adipose tissue not only supply nutrients and oxygen to nourish adipocytes, but also provide cytokines that regulate mass and function of adipose tissue. Understanding the fundamental mechanisms how vessels modulate adipocyte functions would provide new therapeutic options for treatment of metabolic disease and obesity. In recent years, researches about ghrelin are focused on glucose and lipid metabolism, but its effect on vascular function remains uncharacterized. In the present study, ghrelin receptor gene deletion mice (*Ghsr*^−/−^ mice) were used to study ghrelin-regulated vascular metabolism in white adipose tissue. *Ghsr*^−/−^ mice demonstrated lower food intake, lower body weight, and resistance to high-fat diet-induced obesity. The number of vessels in white adipose tissue was decreased in *Ghsr*^−/−^ mice when compared with wild type mice fed with high-fat diet. To further define ghrelin effects in vitro, we used endothelial progenitor cells from wild type and *Ghsr*^−/−^ mice as well as human umbilical vein endothelial cells in our experiments. We found that ghrelin stimulated endothelial cells angiogenesis and migration through the MEK-ERK signaling pathway. [d-Lys3]-GHRP-6 and PD98059 could reverse the effects of ghrelin on endothelial cells. Our study indicates that ghrelin activates its receptor on endothelial cells to promote angiogenesis and migration via a mechanism involving the extracellular regulated protein kinases (ERK) signaling pathway.

## 1. Introduction 

Adipose tissues are highly vascularized, brown adipose tissue (BAT) is one of the most vascularized tissues in the body [1,2,3]. During adulthood, white adipose tissue (WAT) experiences an expansion or shrinkage depending on the energy consumption and metabolic demand of the host [1,4]. 

Vascular system plays a significant role in controlling adipose tissue mass and functions [5,6,7,8]. It controls the fat mass by altering the number of capillaries. Angiogenic vessels not only supply nutrients and oxygen that are required for adipose tissue growth, but also provide growth factors and cytokines to maintain their physiological functions [9,10]. Alterations of WAT mass and functions coordinated with angiogenesis or vascular regression, which are regulated by various types of growth factors, cytokines, and adipokines [7]. Therefore, understanding the molecular mechanisms that control adipose tissue angiogenesis may provide new vascular targets for the treatment of obesity and metabolic disorders [11,12]. 

Ghrelin is a 28-AA peptide hormone that is secreted by X/A-like endocrine cells in the stomach [13]. Recent studies have found that ghrelin and its receptors are widely present in various organs and tissues [14]. Binding to its receptor- growth hormone secretagogue receptor 1a (GHSR1a), ghrelin plays a variety of biological effects, besides promoting the release of growth hormone, ghrelin also stimulates food intake and regulates fat metabolism [15]. As vasoactive peptide, ghrelin also has cardiovascular effects, such as lowering blood pressure, coronary artery expansion, improving heart failure, and a variety of other biological effects [2,16,17]. 

In the present study, we found that except for lower body weight and resistance to high-fat diet-induced obesity, *Ghsr*^−/−^ mice also had fewer vessels in adipose tissue when compared with wild type mice. In vitro experiments demonstrate that ghrelin stimulates angiogenesis and migration of endothelial cells by direct activating its receptor GHSR1a. This effect is mediated via the extracellular regulated protein kinases (ERK) signaling pathway. This study provides evidence that ghrelin plays a very important role in controlling endothelial angiogenesis and migration. In adipose tissue, lacking of ghrelin would decrease vascular density and thus result in smaller adipose tissue mass. 

## 2. Results 

### 2.1. Effects of GHSR1a Blockade on Body Weight and Adipose Tissue Blood Vessels

**Figure 1:**
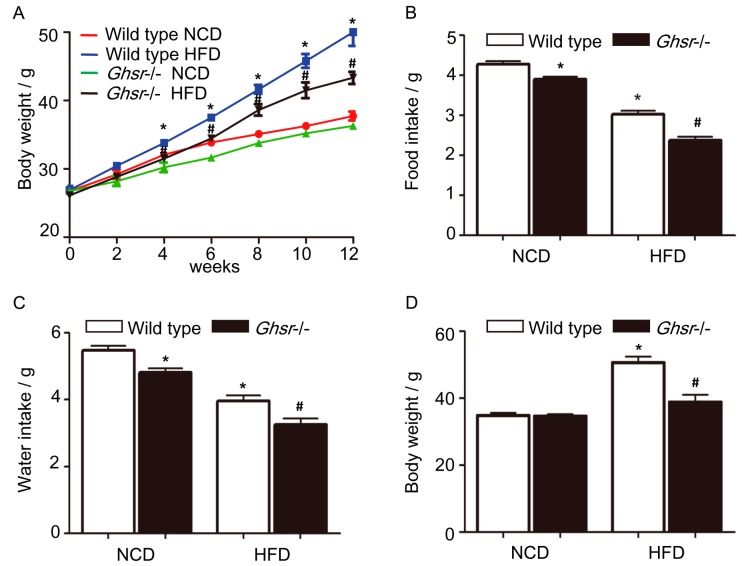

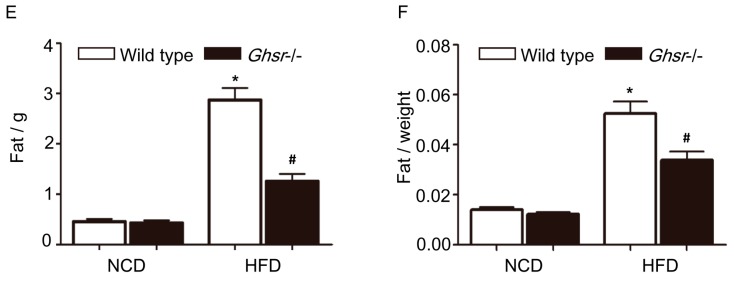
Phenotype of *Ghsr*^−/−^ mice. Wild type or *Ghsr*^−/−^ mice were fed with either normal chow diet (NCD) or 45% high fat diet (HFD) for 12 weeks. (**A**) Body weight change of wild type or *Ghsr*^−/−^ mice fed with NCD or HFD. (**B**–**E**) Average daily food intake (**B**), average daily water intake (**C**), average body weight (**D**) or average weight variation of epididymal adipose tissue (**E**) of wild type or *Ghsr*^−/−^ mice fed with NCD or HFD is shown. (**F**) The epididymal adipose tissue/body weight ratio in wild type or *Ghsr*^−/−^ mice fed with NCD or HFD is shown. Data are expressed as mean ± SD, * denotes *p* < 0.05 compared with wild type mice fed with NCD, ^#^ denotes *p* < 0.05 as compared with wild type mice fed with HFD.

**Figure 2:**
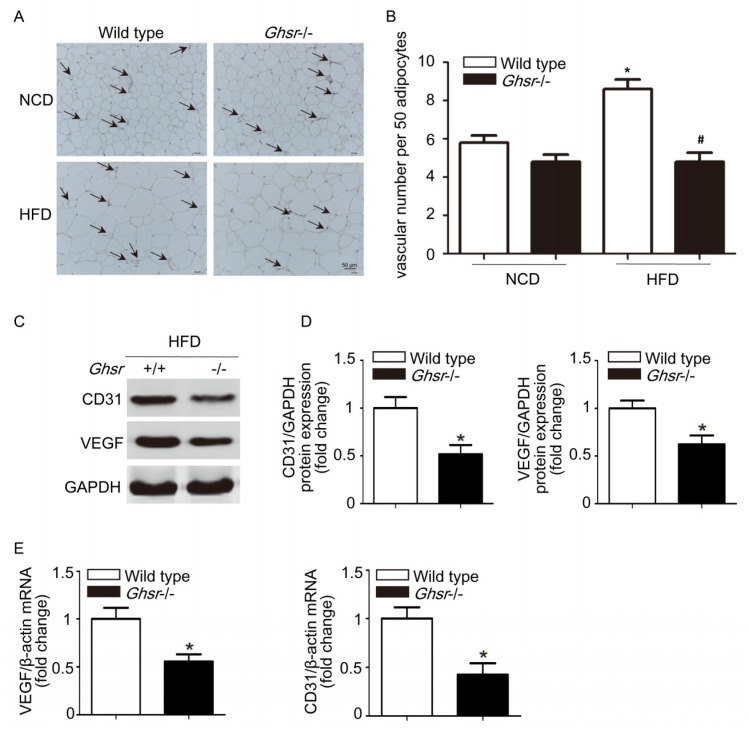
Blood vessels in adipose tissue of *Ghsr*^−/−^ mice and wild type mice. Wild type or *Ghsr1*^−/−^ mice were fed with either NCD or 45% HFD for 12 weeks. (**A**,**B**) Epididymal adipose tissues from wild type mice or *Ghsr*^−/−^ mice were selected and stained with anti-vWF. Results are representative images from seven different animals. The arrows indicate positive staining. Microvessels are counted and averaged in 50 tissues sections. Magnification, ×100. Data are expressed as mean ± SD, * denotes *p* < 0.05 compared with wild type mice fed with NCD, ^#^ denotes *p* < 0.05 as compared with wild type mice fed with HFD. (**C**,**D**) The protein level of CD31, VEGF, or GAPDH was determined by western blotting analysis. (**E**) qRT-PCR analysis of the mRNA level of EC markers. Data are expressed as mean ± SD, * denotes *p* < 0.05.

To determine whether the ghrelin receptor, GHSR1a, had effects on body weight and adipose tissue blood vessels, we used GHSR1a gene knockout mice (*Ghsr*^−/−^ mice) to block the effects of endogenous ghrelin. Wild type mice fed with 45% high fat diet (HFD) for 12 weeks demonstrated significant increases in body weight relative to animals fed normal chow diet (NCD). As shown in Figure 1, *Ghsr*^−/−^ mice were resistant to HFD-induced obesity, with body weight being significantly less than wild type littermates. We used the epididymal white adipose tissue isolated from wild type and *Ghsr*^−/−^ mice fed with NCD or HFD for vWF immunohistochemistry, and found that *Ghsr*^−/−^ mice had fewer blood vessels when compared with wild type mice that were fed with HFD (Figure 2A,B). For further investigation, we examined the vascular endothelial cell (EC) markers in epididymal adipose tissue of *Ghsr*^−/−^ and wild type mice. Western blotting and qRT-PCR analysis showed that *VEGF* and *CD31* expression were decreased in *Ghsr*^−/−^ mice when compared with wild type littermates that were fed with HFD both in protein and mRNA levels (Figure 2C–E).

### 2.2. Effects of Ghrelin Treatment on Angiogenesis and Migration In Vitro 

In the results above, we found that blood vessels were reduced in WAT of *Ghsr*^−/−^ mice compared with wild type mice fed with HFD, suggesting that ghrelin plays a crucial role in angiogenesis. To determine whether ghrelin contributes to EC tube formation and migration, tube-forming ability assay and scratch-wound assay were performed. 

**Figure 3:**
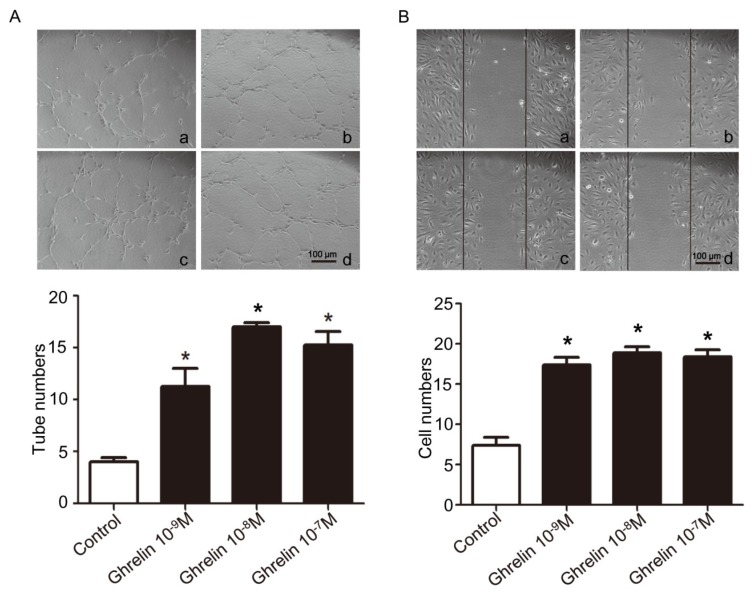

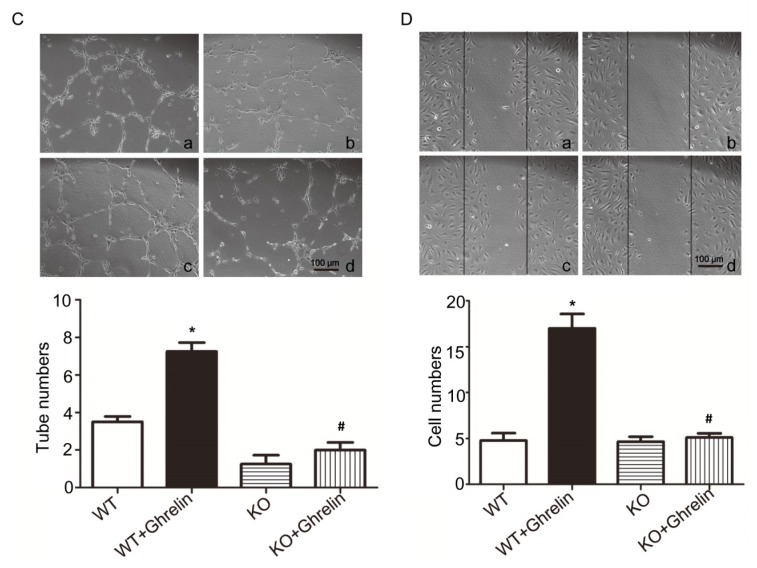
Ghrelin treatment stimulates tube formation and migration of human umbilical vein endothelial cells (HUVECs) and endothelial progenitor cells (EPCs) in vitro. (**A**) In vitro tube formation of HUVECs cultured on Matrigel with ghrelin treatment for 6 h (a, control; b, 10^−9^ M ghrelin; c, 10^−8^ M ghrelin; d, 10^−7^ M ghrelin). Magnification, ×100. Tube-formation assay of HUVEC capillary morphogenesis after ghrelin treatment was shown. The extent of tube formation was determined by counting the tubes in five randomly chosen, low-power fields. (**B**) HUVECs were scratch-wounded and migrating cells were counted after 8 h with ghrelin treatment (a, control; b, 10^−9^ M ghrelin; c, 10^−8^ M ghrelin; d, 10^−7^ M ghrelin), magnification, ×100. Data are expressed as mean ± SD from three independent experiments, each performed in triplicate (* *p* < 0.05 compared with control group). (**C**) In vitro tube formation of EPCs cultured on Matrigel with ghrelin or N.S. treatment for 6 h (a, wild type EPCs + N.S.; b, *Ghsr*^−/−^ (KO) EPCs + N.S.; c, wild type EPCs + 10^−8^ M ghrelin; d, KO EPCs + 10^−8^ M ghrelin), magnification, ×100. Tube-formation assay of EPC capillary morphogenesis after ghrelin treatment was shown. The extent of tube formation was determined by counting the tubes in five randomly chosen, low-power fields. (**D**) EPCs were scratch-wounded and migrating cells were counted after 8 h with ghrelin or N.S. treatment (a, wild type EPCs; b, KO EPCs; c, wild type EPCs + 10^−8^ M ghrelin; d, KO EPCs + 10^−8^ M ghrelin), magnification, ×100. Data are expressed as mean ± SD from three independent experiments, each performed in triplicate, * denotes *p* < 0.05 compared with EPCs from wild type mice without ghrelin administration, ^#^ denotes *p* < 0.05 compared with EPCs from wild type mice with ghrelin administration.

Human umbilical vein endothelial cells (HUVECs) were treated with different concentrations of ghrelin and plated on Matrigel and cultured for 4 h. As is shown in Figure 3A, tube formation increased significantly under ghrelin stimulating. Then, HUVECs were scratch-wounded and migrating cells were counted after 8 h under ghrelin treatment. As shown in Figure 3B, migration capability of HUVECs was also increased by ghrelin treatment.

To further examine the effects of ghrelin, we used endothelial progenitor cells (EPCs) that were isolated from *Ghsr*^−/−^ and wild type mice. EPCs were cultured and identified by flow cytometry. EPCs were treated with 10^−8^ M ghrelin and plated on Matrigel and cultured for 4 h. EPCs were also scratch-wounded and migrating cells were counted after 8 h under ghrelin treatment. As shown in Figure 3C,D, tube formation and migration capability were increased by ghrelin in EPCs from wild type mice, but were not seen in EPCs from *Ghsr*^−/−^ mice.

### 2.3. Effects of Ghrelin on the Phosphorylation of ERK in ECs

As stated above, the effects of ghrelin were mediated by direct activation of its receptor, GHSR1a, and then activating intracellular signaling pathways to achieve [13,18]. Among the various signaling pathways, MEK-ERK pathway is involved in cell proliferation and differentiation, cell morphology and cytoskeleton maintain, and a variety of biological responses [19]. 

**Figure 4:**
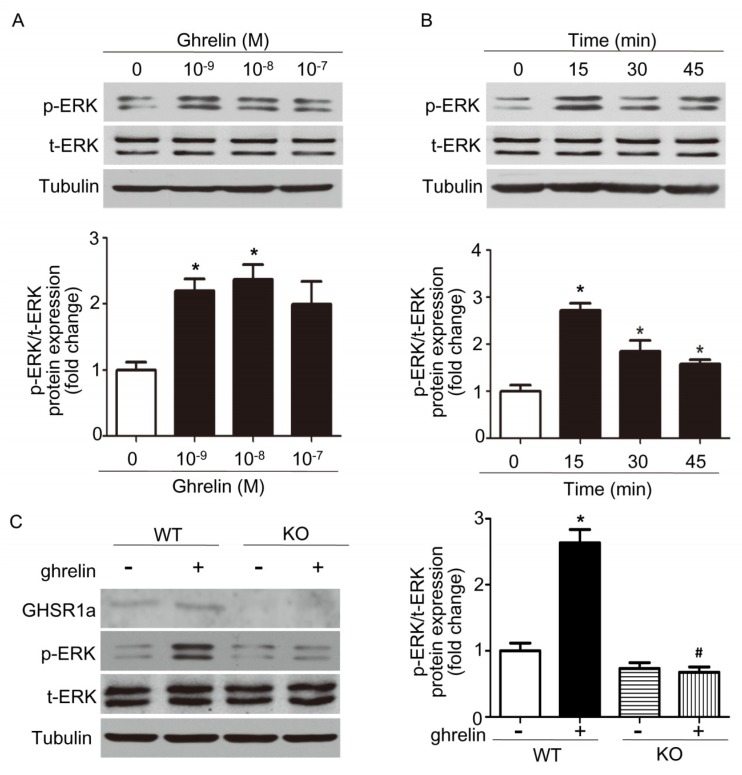
Ghrelin increases the phosphorylation of extracellular regulated protein kinases (ERK) in endothelial cells (ECs). (**A**) HUVECs were incubated with 10^−9^ M, 10^−8^ M, 10^−7^ M ghrelin, or N.S. for 15 min, and the level of phosphorylated ERK or total ERK was determined by western blotting analysis. (**B**) HUVECs were incubated with 10^−8^ M ghrelin or N.S. for 0 min, 15 min, 30 min, or 45 min, and the level of phosphorylated ERK or total ERK was determined by western blotting analysis. Data are expressed as mean ± SD from three independent experiments, each performed in triplicate (* *p* < 0.05 compared with control group). (**C**) EPCs from wild type and *Ghsr*^−/−^ (KO) mice were incubated with 10^−8^ M ghrelin or N.S. for 15 min, and the level of phosphorylated or total ERK or GHSR1a was determined by western blotting analysis. Data are expressed as mean ± SD from three independent experiments, each performed in triplicate, * denotes *p* < 0.05 compared with EPCs from wild type mice without ghrelin administration, ^#^ denotes *p* < 0.05 compared with EPCs from wild type mice with ghrelin administration.

To determine whether ghrelin has effects on the phosphorylation of ERK in ECs, HUVECs were treated with different concentrations of ghrelin for 15 min or treated with 10^−8^ M ghrelin for different time, followed by western blotting to determine the levels of phosphorylated ERK and total ERK. As shown in Figure 4A,B, ghrelin increases the phosphorylation of ERK on a time and concentration-dependent manner. 

We next analyze the effects of ghrelin on EPCs. EPCs isolated from *Ghsr*^−/−^ and wild type mice were incubated with 10^−8^ M ghrelin for 15 min, followed by western blotting to determine the levels of phosphorylated ERK and total ERK. We found that ghrelin increases the phosphorylation of ERK on EPCs from wild type mice, but has no effect on EPCs that were isolated from *Ghsr*^−/−^ mice (Figure 4C). These results showed that the MEK-ERK pathway is involved in tube formation and migration of ghrelin on endothelial cells.

### 2.4. Ghrelin Stimulates Tube Formation and Migration of EPCs through the ERK Dependent Manner

**Figure 5:**
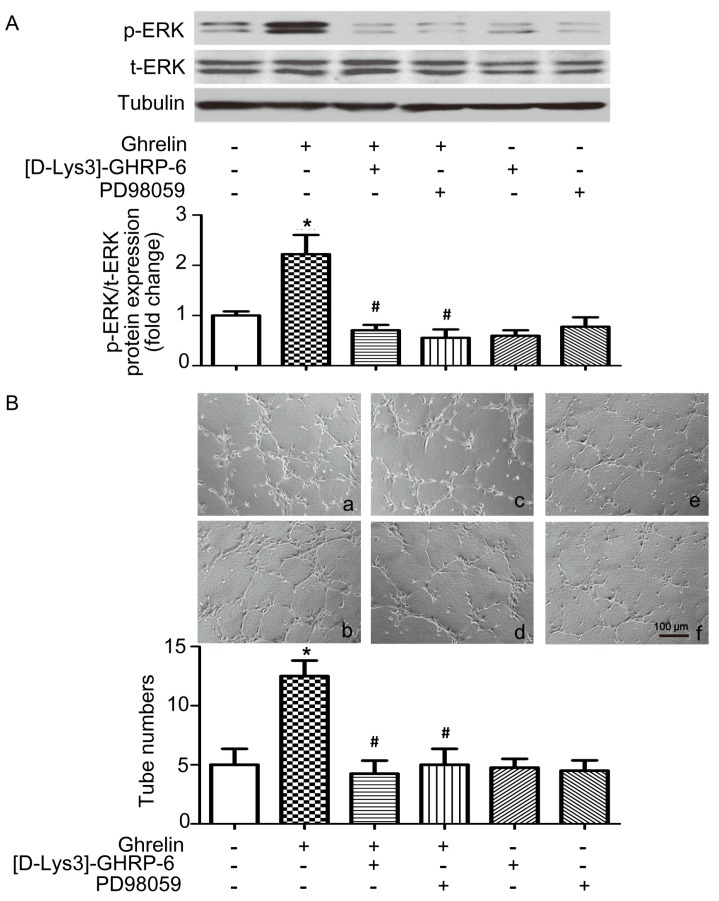

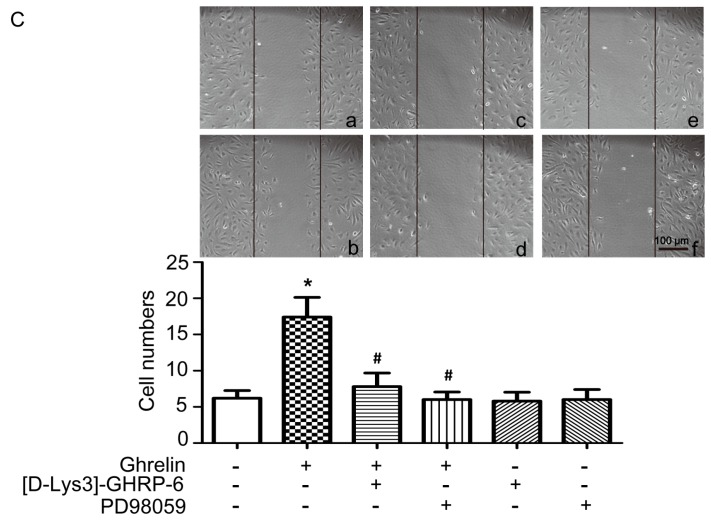
Ghrelin stimulates tube formation and migration of EPCs through the ERK dependent manner. (**A**) Wild type mice EPCs were pre-treated with 100 μM [d-Lys3]-GHRP-6 or PD98059 for 30 min, followed by incubating with 10^−8^ M ghrelin or N.S. for 15 min. The level of phosphorylated ERK or GAPDH was determined by western blotting analysis. (**B**) Tube-formation assay of EPC capillary morphogenesis after ghrelin treatment. In vitro tube formation of EPCs pre-treated with [d-Lys3]-GHRP-6 or PD98059 was performed by cultured on Matrigel with ghrelin or N.S. treatment and cultured for 6 h, magnification, ×100. The extent of tube formation was determined by counting the tubes in five randomly chosen, low-power fields. (**C**) EPCs pre-treated with [d-Lys3]-GHRP-6 or PD98059 were scratch-wounded and migrating cells were counted after 8 h with ghrelin or N.S. treatment, magnification, ×100. a, EPCs + N.S.; b, EPCs + 10^−8^ M ghrelin; c, EPCs + 10^−8^ M ghrelin + 100 μM [d-Lys3]-GHRP-6; d, EPCs + 10^−8^ M ghrelin + 100 μM PD98059; e, EPCs + 100 μM [d-Lys3]-GHRP-6; f, EPCs + 100 μM PD98059. Data are expressed as mean ± SD from three independent experiments, each performed in triplicate, * denotes *p* < 0.05 compared with EPCs without treatment, ^#^ denotes *p* < 0.05 compared with EPCs with ghrelin administration.

The above experiments have verified that ghrelin stimulated phosphorylation of ERK in ECs, to intervene to gain further support of this proposition, EPCs that were isolated from wild type mice were treated with 10^−8^ M ghrelin along with ghrelin receptor antagonist [d-Lys3]-GHRP-6 [20] or MEK-ERK inhibitor PD98059 [21]. As shown in Figure 5A, western blotting analysis showed that the antagonism of ghrelin function or inhibition of MEK-ERK pathway blocks the effect of ghrelin on ERK phosphorylation in EPCs. Furthermore, in vitro assays also showed that antagonism of ghrelin function or inhibition of MEK-ERK blocks the effect of ghrelin on tube formation (Figure 5B) and the migration of EPCs (Figure 5C).

## 3. Discussion

Ghrelin and GHSR1a are widely present in various organs [14]. After binding together, ghrelin plays a variety of biological effects [22]. While multiple studies indicate that ghrelin plays an important role in controlling energy supply [23,24], pathways mediating endothelial cells are less well-described.

In our previous study, we found that ghrelin plays important role in controlling glucose and lipid metabolism [23]. We also noticed that there was a change in adipose tissue blood vessels when intervened ghrelin and its receptor. White adipose tissues (WAT) and brown adipose tissues (BAT) are hyper vascularized. The vascular system plays a significant role in controlling adipose tissue mass and functions [2,9,10,25,26]. Understanding the fundamental mechanisms that vascular modulate adipocyte functions would provide new therapeutic options for the treatment of metabolic disease and obesity.

In order to intervene ghrelin and its receptor, we breed ghrelin receptor deletion mice (*Ghsr*^−/−^ mice) and wild type littermates mice as control. Fed with NCD or HFD for 12 weeks, we examined the vascular phenotype in epididymis adipose tissue. Immunohistochemistry, western blotting, and qRT-PCR assay are used to determine the vascular phenotype. After being fed with HFD, both immunohistochemistry, protein, and mRNA levels indicated that *Ghsr*^−/−^ mice had fewer blood vessels. 

Angiogenesis is the physiological process through which new blood vessels form from pre-existing vessels [27]. Angiogenesis is a normal and vital process in growth and development, which contains extracellular matrix degradation, endothelial cell activation proliferation, and migration, which is a complex process involving multiple molecules of a variety of cell [9,28]. Regression or overexpression of vessels will break the balance with adipose tissue metabolism [15,29]. 

Vascular functions in modulation of adipose tissue weight and insulin sensitivity are divided into two groups of views. One opinion is that the inhibition of vessels in adipose tissue leads to lean phenotype [2,30,31,32,33]. The other one is that acceleration of vessels would lead to lean phenotype in mice [25,34,35].

The initial evidence of vasculatures in modulation of adipose metabolism was detected from ob/ob and HFD induced obese mouse models that received treatment of an angiogenesis inhibitor, TNP-470, which is relatively specific for endothelial cells. TNP-470-treated obese mice had reduced vascular density of adipose along with smaller adipose tissue [2,33]. 

VEGF, which is an endothelial growth factor, plays a vital role controlling vessels in various organs. Temporal repression of systemic VEGF expression leads to a lean phenotype and resistance to diet-induced obesity [33]. On the other hand, several other studies show that overexpression, but not repression, of VEGF in adipose tissues protects against diet-induced obesity and insulin resistance [25,34]. Besides, in metabolic active adipose tissue (BAT), increase the blood vessels would lead to bigger energy consumption, which will inhibit the development of obesity [36,37]. 

However, adipose tissue is one of the largest tissues in the body, the appropriate method of increasing or inhibiting angiogenesis therapy requires close coordination of the organizations and organs with consideration of their metabolic state [6,25,31,33,34,38].

Ghrelin receptor deletion mice (*Ghsr*^−/−^ mice) are resistant to high fat diet [23], have fewer adipose tissue mass [39], and fewer micro blood vessels in it. We detected whether ghrelin influences the process of angiogenesis directly or not. We chose different type of endothelial cells to examine the effects of ghrelin on angiogenesis. In our experiments, ghrelin is highly related to the tube formation and migration of the HUVECs and wild type mice EPCs, which is consistent with the previous reports with cardiac microvascular endothelial cells that were isolated from left ventricular myocardium of adult Sprague-Dawley (SD) rats [40]. When using the EPCs from wild type mice, ghrelin receptor antagonist, or MEK-ERK inhibitor significantly attenuated the effects of ghrelin on angiogenesis. The role of ghrelin receptor in the phosphorylation of ERK signaling pathway was further confirmed with *Ghsr*^−/−^ mice, for ghrelin treatment could not activate ERK phosphorylation of EPCs from *Ghsr*^−/−^ mice. 

In conclusion, our studies suggest that ghrelin is a potent activator of angiogenesis. This finding may explain in part the lean phenotype observed in the *Ghsr*^−/−^ mice fed with high fat diets.

## 4. Materials and Methods 

### 4.1. Ethical Approval

The animals that were used in this study were handled in accordance with the Guide for the Care and Use of Laboratory Animals published by the US National Institutes of Health (NIH publication no. 85–23, revised 1996), and all of the experimental protocols were approved by the Animal Care and Use Committee of Peking University (Permit Number: LA2012-60, approved on 24 February 2012; and LA2016-123, approved on 24 February 2016). The investigators understand the ethical principles under which this journal operates and confirm that the work complies with the journal animal ethics checklist.

### 4.2. Animals and Animal Care

GHSR1a gene knockout mice (*Ghsr*^−/−^ mice) in which exon 1 and exon 2 were deleted were obtained from the Shanghai Research Center for Biomodel Organisms [41]. *Ghsr*^−/−^ mice and wild type mice were housed in standard plastic rodent cages and maintained in a regulated environment (24 °C, 12 h light and 12 h dark cycle with lights on at 0700 and off at 1900). Regular chow and water were available *ad libitum* unless specified otherwise. Where indicated, four-week-old mice were assigned to receive normal chow diet (control diet, D12450H; Research Diets) or a high fat diet (45% fat, D12451; Research Diets) for 12 weeks. Body weight was measured every week. Food and water intake was measured every three days and mean intake per day was calculated. Spillage was weighted and subtracted. Mice were then sacrificed and epidydimal fat pad were taken and weighed.

### 4.3. Human Umbilical Vein Endothelial Cells (HUVECs) Culture, Identification, and Treatment

The investigation confirmed to the principles outlined in the Declaration of Helsinki for use of human umbilical cord blood. The protocol was approved by Peking University Institutional Human Sample Use Committee. Briefly, human cord blood from umbilical cords of new born was collected with the use of heparin (20 U/mL) from donors with their written permission. Human cord blood HUVECs were isolated by density-gradient centrifugation with Ficoll (1.077 g/mL) and plated on dishes that are coated with collagen type I (50 mg/mL; Millipore, Burlington, MA, USA). M199 culture medium was supplemented with 20% FBS, human VEGF (10 ng/mL), human bFGF (1 ng/mL), human EGF (10 ng/mL), IGF II (2 ng/mL), and LIF (10 ng/mL). HUVECs at passages 2–6 were used. 

### 4.4. Isolation and Identification of Mouse Bone-Marrow-Derived Endothelial Progenitor Cells

Mouse bone-marrow-derived endothelial progenitor cell (EPC) isolation, culture, and identification were as previously described [42]. Briefly, EPCs were collected by flushing the femurs and tibias of wild-type or *Ghsr*^−/−^ mice, 8–10 weeks old, with EGM-2 medium containing 10% FBS. Cells were plated on type I collagen-coated dishes and maintained in a humidified atmosphere containing 5% CO_2_ at 37 °C. After 4–7 days in culture, non-adherent cells were removed and adherent cells were cultured for an additional 10 days and then used for further analysis. This population grew at a high proliferation rate into a monolayer of spindle-shaped cells. EPCs at passages 2–4 were used in the following experiments. EPCs were identified as exhibiting high levels of endothelial lineage markers UEA, c-kit and CD31. Moreover, we identified their tube-forming function by their pro-angiogenic capacity to form tube-like structures on Matrigel [43].

### 4.5. Protein Extraction and Western Blotting

Western blot analysis was carried out according to standard protocols. Cultured cells were harvested and homogenized in ice-cold fractionation buffer containing RIPA lysis buffer, Phenylmethanesulfonyl fluoride (PMSF), and protein phosphatase inhibitor mixture. The cell lysate was treated with ultrasound for 3 s three times, and then centrifuged at 12,000 rpm for 10 min at 4 °C. After centrifugation, the supernatant was used for Western blot analysis. Protein concentration was measured by the Bradford’s method. A total of 40~60 μg protein from each sample was loaded. Proteins were transferred to polyvinylidene fluoride membranes. The membranes were incubated for 1 h at room temperature with 4% fat-free milk in Tris buffered saline containing Tween-20, followed by incubation overnight at 4 °C with the individual primary antibody. Specific reaction was detected using IRDye conjugated second antibody and visualized while using the Odyssey infrared imaging system (LI-COR Biosciences, Lincoln, NE, USA). Quantification of image density in pixel was performed using the Odyssey infrared imaging system (LI-COR Biosciences, Lincoln, NE, USA). Primary antibodies used were anti-p-ERK, and anti-ERK (1:1000; Cell Signaling, Beverly, MA, USA); anti-vWF, anti-GHSR1a, and anti-VEGF (1:1000; Santa Cruz Biotech nology, Santa Cruz, CA, USA); and, anti-GAPDH and anti-β-actin (1:1000; Abcam, Cambridge, MA, USA).

### 4.6. Quantitative Real-Time Polymerase Chain Reaction (qRT-PCR)

Total RNA was isolated while using the TRIzol reagent (Invitrogen, Carlsbad, CA, USA). Reverse transcription was performed using the RT system according to the manufacturer’s instruction. PCR was conducted in a 25 μL volume containing 2.5 μL cDNA, 5 mM MgCl_2_, 0.2 mM dNTPs, 0.2 μM each primer, 1.25 U AmpliTaq Polymerase, and 1 μL 800× diluted SYBRGreen I stock while using the Mx3000 multiple quantitative PCR system (Strata gene, La Jolla, CA, USA). PCR reactions were performed in duplicate. Primers used in this study are shown in Table 1. All the mRNA expression was quantified using the comparative cross threshold (*C*t) method. The *C*t value of the housekeeping gene β-actin was subtracted from the *C*t value of the target gene to obtain Δ*C*t. The normalized fold changes of target gene mRNA expression were expressed as 2^−ΔΔ*C*t^, where ΔΔ*C*t equals to Δ*C*t sample −Δ*C*t control.

### 4.7. Flow Cytometry

Fluorescence-activated cell sorting (FACS) analysis was used to detect cell surface markers. Cells were stained for 60 min at 48 °C, then fixed with 2% paraformaldehyde. The surface markers investigated were FITC-conjugated mouse anti-human UEA, FITC-conjugated mouse anti-human CD31, and Alex-488-conjugated mouse anti-human c-kit (all from BD PharMingen, Franklin Lakes, NJ, USA). Isotype-identical antibodies served as negative controls. Analysis involved the use of FACS Calibur (Becton, Dickinson and Company (BD), Franklin Lakes, NJ, USA) and Cell Quest software.

### 4.8. In Vitro Tube-Formation Assay

Matrigel (300 μL; Becton Dickinson, Bedford, MA, USA) was added to each well of 24-well plates. HUVECs in M199 medium and EPCs in EBM-2 medium supplemented with 20% FBS were then plated at 1 × 10^5^ cells/well and then cultured for 4 h. After treatment with various stimuli, tube formation was examined while using an inverted microscope equipped with a digital camera. Tube-like structures exceeding six cells in length were counted in five randomly selected fields in each well by three investigators blinded to the treatment.

### 4.9. Cell Migration

HUVEC and EPC migration was assessed by scratch-wound assay, as described previously [44]. A wound was created in the cell monolayer, and images of cells were captured then and 8 h later. Images were quantified to determine the number of migrating cells.

### 4.10. Histology Analysis and Immunofluorescence

Formalin-fixed tissues were embedded in paraffin and sectioned 4 μm thick. Hematoxylin and eosin (H&E) staining was carried out, as described before [45]. For immunofluorescence, antigen retrieval was processed after rehydration in citrate acid buffer (pH 6.0) at 95 °C for 15 min. Sections were blocked with 5% BSA in PBS for 1 h at room temperature and then incubated overnight with anti-CD31 primary antibody diluted at 1:100 at 4 °C (Santa Cruz, Santa Cruz, CA, USA) and fluorescein iso-thiocyanate secondary antibody (Invitrogen) diluted at a 1:1000 for 2 h at room temperature, followed by 4 °C, 6-diamidino-2-phenylindole staining for nuclei. PBS washes were performed between incubations. Sections were kept in the dark from the beginning of secondary antibody incubation and finally mounted with glycerol and immediately analyzed by fluorescent microscope (Nikon, Tokyo, Japan).

### 4.11. Statistical Analysis

Data were expressed as means ± SD. Data analysis used GraphPad Prism software. One-way ANOVA, Student-Newman-Keul’s test (comparisons between multiple groups), or unpaired Student’s *t* test (between two groups) was used as appropriate. *p* value < 0.05 denotes statistical significance.

## Figures and Tables

**Table 1:** List and sequence of primers.

	Upstream Primer (5′–3′)	Downstream Primer (5′–3′)
*GHSR1a*	CTATCCAGCATGGCCTTCTC	AAGACGCTCGACACCCATAC
*VEGF*	GATCATGCGGATCAAACCTC	AATGCTTTCTCCGCTCTGAA
*CD31*	ATGATGCCCAGTTTGAGGTC	GACGTCTTCAGTGGGGTTGT
*β-actin*	ATCTGGCACCACACCTTC	AGCCAGGTCCAGACGCA
*GAPDH*	ATGACATCAAGAAGGTGGTG	CATACCAGGAAATGAGCTTG
